# Fabrication and characterization of a pH‐sensitive intelligent film incorporating dragon fruit skin extract

**DOI:** 10.1002/fsn3.2680

**Published:** 2021-12-15

**Authors:** Nurnabila Afiqah Azlim, Abdorreza Mohammadi Nafchi, Nazila Oladzadabbasabadi, Fazilah Ariffin, Pantea Ghalambor, Shima Jafarzadeh, A. A. Al‐Hassan

**Affiliations:** ^1^ Food Technology Division School of Industrial Technology Universiti Sains Malaysia Penang Malaysia; ^2^ Department of Food Science and Technology, Damghan Branch Islamic Azad University Damghan Iran; ^3^ Department of Food Science and Technology, Science and Research Branch Islamic Azad University Tehran Iran; ^4^ School of Engineering Edith Cowan University Joondalup Western Australia Australia; ^5^ Department of Food Science and Human Nutrition College of Agriculture and vit. Medicine Qassim University Burydah Saudi Arabia

**Keywords:** anthocyanin, gelatin, intelligent packaging, pH indicator

## Abstract

A novel intelligent pH‐sensing indicator based on gelatin film and anthocyanin extracted from dragon fruit skin (*Hylocereus polyrhizus*) (DFSE) as a natural dye was developed to monitor food freshness by the casting method. Anthocyanin content of DFSE was 15.66 ± 1.59 mg/L. Dragon fruit bovine gelatin films were characterized by Fourier transform infrared spectroscopy (FTIR) and observed by a scanning electron microscope (SEM). Moisture content, mechanical properties, water solubility, water vapor permeability (WVP), light transmittance, color, and pH‐sensing evaluations were evaluated for potential application. FTIR spectroscopy revealed that the extracted anthocyanin could interact with the other film components through hydrogen bonds. When the extract was added, films showed a smooth and clear surface as observed by SEM. The addition of anthocyanin increased the moisture content, thickness, and water solubility of the films, but decreased the WVP and light transmittance of films. Also, the incorporation of 15% v/v DFSE decreased the tensile strength from 17.04 to 12.91 MPa, increasing the elongation at break from 91.19% to 107.86%. The films showed higher Δ*E* with increasing DFSE content, which indicated that the film had good color variability. A significant difference in the color of the films was observed with exposure to different pH buffer solutions. The findings demonstrated that gelatin film incorporated with DFSE could be used as a visual indicator of pH variations to monitor the freshness of foods during storage time.

## Graphical abstract

A novel intelligent pH‐sensing indicator based on gelatin film and anthocyanin extracted from dragon fruit skin (*Hylocereus polyrhizus*) (DFSE) as a natural dye was developed. Gelatin film incorporated with DFSE could be used as a visual indicator of pH variations to monitor the freshness of foods during storage time.

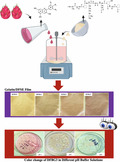

## 1 INTRODUCTION

In the last few years, intelligent packaging development to increase consumer concerns about food safety and quality has been investigated (Chi et al., 2020; Dong et al., 2020). They have become popular since the packaging components can monitor the food and environment surrounding it (Chen et al., 2018). Intelligent packaging method simplifying decision‐making to maintain food quality, prolong shelf life, and improve overall food safety. It can convey intelligent purposes, such as detecting, identifying, and recording certain kinds of information (Kang et al., 2020). Therefore, intelligent packaging systems involve hardware mechanisms such as time–temperature indicators, gas detectors, freshness, and/or ripening indicators (Andretta et al., 2019; Salarbashi et al., 2021; Zhai et al., 2019). Indicators notify about a detected variation in a product or its surroundings, for example, a change in temperature or pH level. In food packaging, biosensors are often used to discover, record, and transfer evidence related to the possible biological processes and responses (Soon & Manning, 2019).

Mainly, pH‐based colorimetric sensing films can be used to express information on food freshness (Zhai et al., 2017; Zhang et al., 2020). The pH‐indicator films are intelligent packaging films that have gained wide attention as they display information by a distinct color change. Generally, pH‐indicator films comprises sensitive dyes and the solid matrix. The chemosynthetic dyes such as xylenol blue, methyl red, bromocresol green, and bromophenol blue are sensitive dyes used to develop the pH‐indicator film (Morsy et al., 2016). Nevertheless, chemosynthetic dyes have potential pathogenicity and toxicity (Zhang et al., 2014). Anthocyanins are a suitable alternative dye because they are nontoxic, safe, and friendly to the environment and the human body. Anthocyanins are a group of phenolic compounds belonging to the flavonoid family responsible for the blue, purple, and red hues of plant leaves, flowers, and fruits (Chen et al., 2020; Wu et al., 2019). The incorporation of anthocyanins in the intelligent film is natural, and these are water‐soluble phenolic compounds and nontoxic (Zhai et al., 2017). The structural transformations of anthocyanins are associated with a color change in pH function (Singh et al., 2018). Incorporating anthocyanin, which plays an essential role as a natural dye in the film, can display the food condition (Xue Mei et al., 2020; Zhai et al., 2017). Besides, anthocyanins are very sensitive to pH and temperature, quickly changing their color upon exposure to a specific environment (Dong et al., 2020). Therefore, food spoilage can be observed from the color change of the films due to the presence of anthocyanin inside the film (Zhang et al., 2020).

Many research types have produced intelligent films with anthocyanin from various sources (Singh et al., 2018). Tichoniuk et al. (2017) reported an edible film, which consists of pH‐sensitive dye, that can be prepared easily and has excellent sensitivity to pH and temperature.

Dragon fruit skin contains a significant amount of anthocyanin and is highly nutritive (Vargas et al., 2013). Recently, attempts have been made to use this natural color as an alternative to synthetic dyes used in the food (Thirugnanasambandham & Sivakumar, 2017).

Smart films contain a solid matrix, which is mainly fabricated by a degradable polymer. Researchers have turned their attention to natural polymers such as starch, gelatin, and fish protein (Arezoo et al., 2020; Hanani et al., 2011; Jafarzadeh et al., 2021; Jafarzadeh et al., 2021; Lin et al., 2019). Due to its excellent film‐forming ability (Abedinia et al., 2020; Al‐Hassan et al., 2021; Hashemi Tabatabaei et al., 2018), gelatin has gained importance.

Alpaslan et al. (2020) produced films from pomegranate extract that can exhibit the spoilage of pasteurized whole milk and cheese by changing the film color. Anthocyanin from Amaranthus leaf extract can be used to monitor the freshness of chicken meat (Kanatt, 2020). Also, the fabricated intelligent film with the addition of anthocyanin from roselle can detect the freshness of fish (Zhai et al., 2017). Studies have detected squid spoilage by using anthocyanin as a pH indicator (Ahmad et al., 2018; Tomac & Yeannes, 2012).

Most consumers prefer high‐quality, smart packaging and adding anthocyanin from dragon fruit skin into intelligent films can fulfill this demand. This study aimed to develop and characterize a pH‐sensitive gelatin‐based intelligent film by incorporating anthocyanin extracted from dragon fruit skin. The influence of the DFSE content on mechanical and physical properties was investigated. Furthermore, the sensing ability of intelligent films to different pH, scanning electron microscopy (SEM), Fourier‐transform infrared (FTIR) spectroscopy, followed by light transmittance measurements, was also examined.

## 2 MATERIALS AND METHODOLOGY

### 2.1 Materials

Bovine gelatin was purchased from SIM Supply Company. Glycerol and salts to prepare required relative humidity (RH) and buffer solutions for anthocyanin were of analytical grade. Dragon fruits were obtained from the local market (Perak, Malaysia).

### 2.2 Extraction of anthocyanins from dragon fruit skin (DFSE)

Anthocyanins were extracted from dragon fruit skin as described by Luchese et al. (2017), with a slight modification. The dragon fruit skin was washed and peeled off before the skin was cut into small pieces. Then, the dragon fruit skin was placed in a freezer (−2°C) overnight. The skin of the dragon fruit was freeze‐dried for 3 days, milled, and sieved to get the final powder. The 200‐mesh of dragon fruit skin powder was collected for extraction of anthocyanin. Anthocyanins were extracted from DFS, according to Wu et al. (2020), with some modifications. Anthocyanins were extracted by immersing the powder in 100 ml of 80% (v/v) ethanol solution. Five grams of the powder was weighed and poured into a 100 ml mixture of ethanol and distilled water. The solution was stirred for 1 h under 500 rpm. Thereafter, the solution was filtered using Whatman filter paper No. 3, and the extracted anthocyanin content was kept in a dark bottle at a temperature of 4°C.

### 2.3 Total anthocyanin content in DFSE

The anthocyanin content in dragon fruit skin was analyzed using the pH differential method (Sengkhamparn et al., 2013). Briefly, the sample was dissolved in buffer solution pH 1 (0.25 M potassium chloride and HCl) and pH 4.5 (0.4 M sodium acetate). The absorbance of the solution in each pH buffer was measured at 510 and 700 nm. The determination was performed in triplicate. The concentration of anthocyanin was then calculated as follows:

$$\text{Anthocyanin}\;\left(\frac{\text{mg}}{L}\right)=\frac{A\text{.MW}\text{.DF}.1000}{\varepsilon .L}$$

where MW is the molecular weight of anthocyanin (449.2) for cyanidin‐3‐glucoside, DF is a dilution factor, *L* is the path length, *ε* is the molar absorptivity (26,900), and *A* is the absorbance of the sample, which was calculated as shown below:

$$A=\left(A_{510}‐A_{700}\right)\;\text{pH}\;1.0‐\left(A_{510}‐A_{700}\right)\;\text{pH}\;4.5$$

### 2.4 Preparation of dragon fruit/bovine gelatin (DFBG) films

Dragon fruit/bovine gelatin film was fabricated using the method of Abdorreza et al. (2011) with slight modifications. An amount of 30% glycerol (w/w, dry basis) was added into 100 ml distilled water before slowly adding 4% w/v of gelatin. Thereafter, the extract from DFS was added at different amounts: 0% (DFBG0), 5% (DFBG1), 10% (DFBG2), and 15% v/v (DFBG3). The solution was stirred continuously for approximately 40–45 min at 45°C. The film solution was then cast into 16 cm × 16 cm frame and dried at 40°C for 24 h.

### 2.5 Characterization of DFBG films with different concentrations of DFSE

#### 2.5.1 Moisture content

According to the method described by Pourjavaher et al. (2017), the samples were prepared in triplicate with different lengths of 5 × 15 mm^2^, 5 × 20 mm^2^, and 5 × 30 mm^2^, each being conditioned in a bottle at a controlled room temperature of 23°C and 55% RH for 2 days and dried in an oven (105°C) overnight. The moisture content was then determined by using the following formula:

$$\text{Moisture}\;\text{content}\;\left(\%\right)=\frac{W_{i}‐W_{t}}{W_{i}}\times 100$$

where *W*_t_ is the weight of film after drying (g) and *W*_i_ is the initial weight (g) after conditioning.

#### 2.5.2 Mechanical properties

The elongation at break (EB%) and tensile strength (TS) of sample films were determined using the auto tensile tester TA‐TX2 texture analyzer (Stable Micro System) with a strain rate of 30 cm/min according to ASTM D882‐16. Before testing, the films were cut into rectangular strips (2 cm × 10 cm) and conditioned at 25°C and 53% RH environment for 48 h. The film's thickness was measured using a digital micrometer (ID‐C112XBS, Mitutoyo Corp.). For each film, five random positions were used for measurement.

#### 2.5.3 Water vapor permeability

Water vapor permeability (WVP) was determined gravimetrically according to ASTM E96‐16 standard method with slight modifications. The films were cut into a circular shape, and the thickness was measured with a micrometer (Mitutoyo Corp.) at seven randomly selected points. Each of the films was then sealed to the open mouth of gas permeation cells containing silica gel. The films were sealed with parafilm on top of the cells. These cells were then placed in a desiccator. Thereafter, the water vapor movement rate through the film into the silica gel was determined by periodic daily weighing over 7 days. The water vapor transmission rate determined by weight gain of the cells. A graph of weight gain as a function of time was plotted. Water vapor transmission rate (WVTR) was calculated from the slope of the graph divided by the permeation area. The sample was prepared in duplicate for each film. WVP was calculated as follows:

$$\text{WVP}=\frac{\text{WVTR}}{S({\text{RH}}_{1}‐{\text{RH}}_{2})}\;\times \;d$$

where *S* is the saturated water vapor pressure at test temperature (Pa), RH_1_ is the relative humidity for desiccators, RH_2_ is the relative humidity for permeation cell, and *d* is the film thickness (*m*).

#### 2.5.4 Water solubility

The water solubility was evaluated according to the method reported by Dong et al. (2020) with some modifications. Rectangular‐shaped (3 cm × 2 cm) pieces of films were dried in an oven at 100°C for 24 h to determine the initial dry weight (*W*_i_). Each sample was then placed in 50 ml of distilled water at room temperature for 1 h, under stirring at 100 rpm. Thereafter, the sample was taken out from the chamber, and the surface water was removed with a filter paper. The wet film sample was then dried in an oven, as mentioned earlier, and the final dry weight (*W*_f_) was obtained. The water solubility of the film was measured with three replicates. WS (%) was calculated by the following equation:

$$\text{WS}\;\left(\%\right)=\frac{W_{f}‐W_{i}}{W_{i}}\times 100$$

where *W*_i_ and *W*_f_ are the initial and final weight of films (dry basis), respectively.

#### 2.5.5 Moisture sorption isotherms

The moisture sorption isotherm of the films was determined through a slightly modified method (Muangrat & Nuankham, 2018). The films were cut into different sizes, pre‐dried in a drying oven for several days, and then accurately weighed. The films were then stored in separate desiccators at specific humidities of 90%, 75%, 53%, 43%, 33%, 22%, and 11% for several days at room temperature to equilibrate. The weight changes of the films were tested to obtain moisture sorption, which was predicted by using the Guggenheim Anderson Boer water vapor sorption model (GAB). Furthermore, the moisture content on a dry basis was calculated to determine the moisture adsorption of films as follows:

$$\text{Moisture}\;\text{content}\;\text{on}\;a\;\text{dry}\;\text{basis}\;\left(\%\right)=\frac{\left(W_{t}‐W_{i}\right)}{W_{i}}\times 100$$

where *W*_t_ is the weight of film at a specific relative humidity (g) and *W*_i_ is the initial weight of dried film (g).

#### 2.5.6 Colorimetric analysis

The color parameters were measured using a Konita Minolta Spectrophotometer colorimeter (CM‐3500d), according to Ma et al. (2017). *L* (lightness), *a* (redness‐greenness), and *b* (yellowness‐blueness) were measured to evaluate the color difference of the films. The sensitivity of the films to acid and alkali solution was measured in an aqueous solution (pH = 4, 7, and 9). The films were first cut and immersed in the aqueous solution for 5 s. After removing the solutions, each film was placed on a Petri dish, and its color was noted without drying. Three measurements were taken on each film. The total color difference (Δ*E*) was calculated as shown below:

$$\Delta E={\left(\Delta L^{*2}+\Delta a^{*2}+\Delta b^{*2}\right)}^{1/2}$$

where $\Delta L^{*}=L‐L_{0}^{*}$; $\Delta a^{*}=a‐a_{0}^{*}$; $\Delta b^{*}=b‐b_{0}^{*}$; and $L_{0}^{*}$, $a_{0}^{*}$, and $b_{0}^{*}$ are the color values of the reference.

#### 2.5.7 pH‐sensing evaluations

The color response of the films was evaluated according to a previous study (Yong et al., 2019) with some modifications. The film (30 mm × 15 mm) was immersed in different pH buffer solutions with pH from 2 to 7. The film color was then measured at three random points by a colorimeter after removing the buffer solutions. The parameters of L*, a*, and b* were recorded to evaluate the film's color. ∆*E* was calculated from an equation as shown below (Rukchon et al., 2014) (if ∆*E* > 3.5, a clear color difference could be noticed; Halász & Csóka, 2018):

$$\Delta E={\left[{\left(L^{*}‐L_{c}^{*}\right)}^{2}+{\left(a^{*}‐a_{c}^{*}\right)}^{2}+{\left(b^{*}‐b_{c}^{*}\right)}^{2}\right]}^{1/2}$$

$L_{c}^{*}$, $a_{c}^{*}$, and $b_{c}^{*}$ are the original color parameters of the film; they are recorded as L*, a*, and b* after sensing, respectively.

#### 2.5.8 Light transmittance

All films were cut in the dimension of the equipment cell (5 mm × 40 mm rectangles) and placed directly in the location of the equipment cell. Thereafter, the UV–visible and absorption spectra of films were determined at 200–800 nm using a UV–Vis spectrophotometer (UV‐2600, Shimadzu), with a blank glass plate as a reference at room temperature (Marvizadeh et al., 2017).

#### 2.5.9 Fourier transform infrared spectroscopy

The FTIR spectra of gelatin‐based films were analyzed using a spectrometer (Perkin Elmer), operating with attenuated total reflectance (ATR) Fourier transform mode. The spectra of the films were measured between 600 and 4000 cm^−1^ wavenumber range and recorded at 4 cm^−1^ resolutions.

#### 2.5.10 Scanning electron microscope

The micrograph of films with different DFSE was examined using SEM (Quanta 200; Philips‐FEI Co.). The samples were coated with a thin layer of platinum before the analysis was carried out at an accelerating voltage of 10 kV with 15 k magnification power.

#### 2.5.11 Statistical analysis

All determinations were performed in triplicate. The data are reported as mean ± standard deviation and analyzed using one‐way analysis of variance (ANOVA) in SPSS (v17.0; SPSS Inc.). The differences between means were carried out by Duncan's multiple range test (*p* < .05).

## 3 RESULTS AND DISCUSSION

### 3.1 Total anthocyanin content

The total anthocyanin content extracted from DFS was calculated by using a pH differential method. The results obtained were significantly lower compared with that obtained in a previous study due to the method used and the flavonoid compounds in the fruit itself (Chaiyasut et al., 2016). The total anthocyanin content in DFS was 15.66 ± 1.59 mg/L. Although the result was relatively low, the anthocyanin content can change color when exposed to different pH solutions. A study conducted by Purbaningtias et al. (2017) also found that the total anthocyanin content was 22 mg/L in red dragon fruit.

On the contrary, some researchers showed a higher total anthocyanin content in red dragon fruit (Chaiyasut et al., 2016). The different results might be due to the extraction method and the method used to calculate the total anthocyanin content.

In fact, anthocyanin content was easily degraded, which might have contributed to the low results. Anthocyanin was very sensitive to pH, light, and temperature. Since it is highly reactive, it quickly degrades or reacts with other constituents in the film matrix to form colorless or brown‐colored compounds (Chaiyasut et al., 2016). In this study, the total anthocyanin content was conducted at room temperature without UV light exposure. Higher results obtained by Purbaningtias et al. (2017) at the higher temperature indicated that the temperature influenced the total anthocyanin content.

### 3.2 Effects of DFSE on physicochemical and mechanical properties of gelatin films

The results of the moisture content for each film concentration are presented in Table 1. The moisture content was found to be not statistically different between samples. This might be because the amount of glycerol used during the preparation of the films was the same for all the formulations. Glycerol, known as a plasticizer, could have affected the moisture content of the films. It can form hydrogen bonds that enable the surface to attract water in the film matrix. According to Golasz et al. (2013), the moisture content was not statistically different between samples because the amount of glycerol added for each film concentration was the same.

**TABLE 1:** Effects of DFSE concentration on mechanical properties of gelatin films

Films	Thickness (mm)	Tensile strength (MPa)	Elongation at break (%)	Water solubility (%)	Moisture content (%)
DFBG0	0.13 ± 0.00^a^	17.04 ± 0.98^a^	91.19 ± 7.67^a^	30.63 ± 9.47^a^	0.24 ± 0.03^a^
DFBG1	0.14 ± 0.01^a^	15.04 ± 0.54^b^	102.53 ± 3.28^a^	35.19 ± 9.32^a^	0.27 ± 0.00^a^
DFBG2	0.15 ± 0.01^b^	13.22 ± 1.08^bc^	104.94 ± 1.84^a^	47.53 ± 4.52^a^	0.28 ± 0.00^a^
DFBG3	0.17 ± 0.00^c^	12.91 ± 1.23^c^	107.86 ± 7.73^a^	52.73 ± 7.98^b^	0.28 ± 0.01^a^

Note

Values are expressed as mean ± standard deviation. Values with the same superscript letter within a column are not significantly different (*p* > .05).

DFBG3 showed the highest moisture content among those films due to the highest amount of anthocyanin added during the film preparation. The anthocyanin from dragon fruit has interrupted the network bond inside the film matrix, increasing the moisture content (Zhang et al., 2019).

The thickness, tensile strength, elongation at break, and water solubility are shown in Table 1. The thickness of DFBG0, DFBG1, DFBG2, and DFBG3 films was 0.134, 0.142, 0.153, and 0.174 mm, respectively. The results showed an increase in thickness since the amount of DFSE incorporated into the film was increased. Thus, it influenced the thickness of the film. However, each film thickness difference was not slightly different since the other additives (glycerol and gelatin) were weighed at the same amount except for the anthocyanins. Putra et al. (2019) demonstrated that anthocyanin from DFSE had increased the total solids in the solution, increasing the thickness of the intelligent film.

The results indicated that the tensile strength of films decreased. There was a significant difference compared with the DFBG0 film and the other film concentration. This might lead to the anthocyanin forming hydrogen and covalent bonds with amino and hydroxyl groups of the polypeptide in gelatin, weakening the protein–protein interactions to stabilize the protein network in the film matrix (Li et al., 2014). Furthermore, the amount of extracted anthocyanin incorporated into the film produced a heterogeneous structure; thus, the tensile strength was lower.

The elongation at break of the films were not significantly different between samples, but a small increase in elongation at break of the films, indicating that DFSE improves flexibility of the films.

Similar results were reported by Qin et al. (2020) for commercially used high‐density polyethylene film and polyvinylidene chloride film. Hence, reduced tensile strength with increased elongation at break produced good film properties similar to synthetic packaging.

Water solubility is one of the most critical parameters that need to be analyzed for the film's sensitivity and its effect on packaging applications. Hydrophilic and hydrophobic components positively influence the packaging material (Ulfah et al., 2018). In this research, the water solubility increased from 30.63% to 52.73% with an increasing concentration of the DFBG.

As shown in Table 1, DFBG3 film showed the highest solubility, which might be due to the presence of hydrophilic compunds in the film matrix. It also demonstrated that it has a significant difference when compared with other film concentrations. Dong et al. (2020) claimed that glycerol in the film might also increase water solubility. It is good to have low water solubility in intelligent films because it can ensure the integrity of the product; however, we found higher water solubility, which indicates that the resistance of this intelligent packaging toward water is low. Besides, the incorporation of DFSE in the intelligent film affects water solubility, which increased the flexibility of DFBG films. This is because it has a high amount of hydrophilic components, which give rise to water solubility.

According to Dong et al. (2020), the materials used during film preparation play an important role, affecting the mechanical properties of the film. The mechanical properties of the film are very crucial to consider when producing better high‐quality food packaging.

### 3.3 Water vapor permeability of gelatin films containing DFSE

The WVP of intelligent films is critical since it significantly affects food shelf life. The water might be transferred from either the internal or external environment through the polymer package wall. Hence, it should be as low as possible to avoid moisture transfer between the food and the surrounding atmosphere (Arham et al., 2016).

**FIGURE 1:**
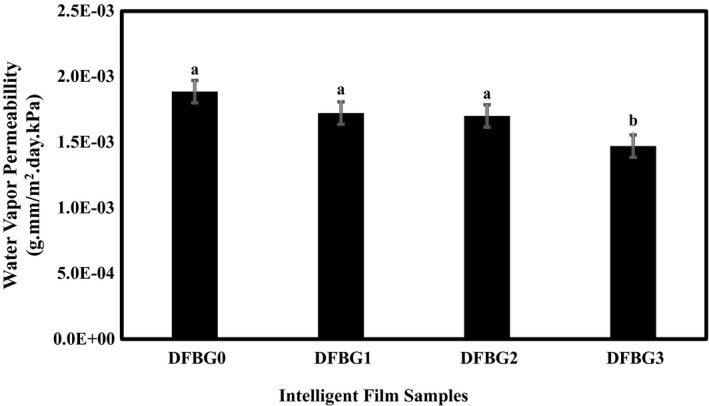
Effects of DFSE concentration on water vapor permeability of gelatin films

In this study, the WVP results of DFBG films decreased with increasing DFSE. Also, the incorporation of DFSE improved the water vapor barrier property of films. As shown in Figure 1, the DFBG3 film showed the lowest WVP value (1.4670 g.mm/m^2^.day.kPa) compared with DFBG1 and DFBG2 films, which were 1.7240 and 1.6927 g.mm/m^2^.day.kPa, respectively. In contrast, as no DFSE was added to DFBG0 film, it showed higher WVP (1.8804 g.mm/m^2^.day.kPa), it is likely due to no interruption of the network of the film matrix that only gelatin and glycerol were used in the film. The changeing trend in the WVP was same as moisture content trend, which was related to the hydrogen bonds in the film matrix (Cazón et al., 2017).

The lower WVP of the films incorporated with anthocyanin from DFSE could be because of hydrogen and covalent bonds forming with the polar groups, resulting in a less hydrophilic layer (Ee et al., 2014). Similar results were obtained when the addition of beetroot residue powder reduced the WVP of gelatin‐based films (Iahnke et al., 2016).

### 3.4 Effects of DFSE on Moisture sorption isotherm of gelatin films

**FIGURE 2:**
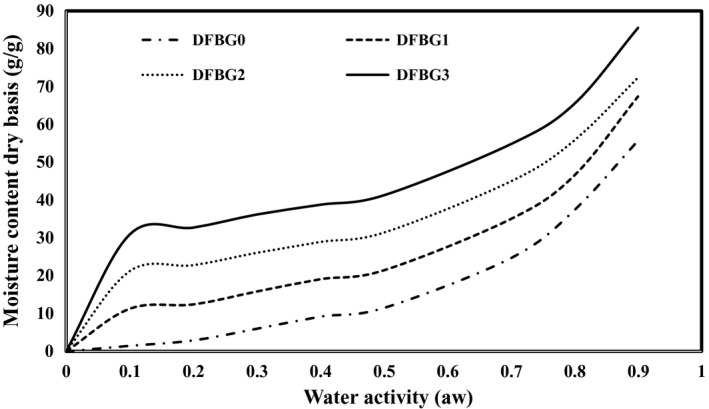
Moisture sorption isotherm of DFBG films

The moisture sorption isotherms of the DFBG films are shown in Figure 2. All films presented sigmoidal curvatures (S‐shaped), which exhibited a slow initial increase in moisture content at a low water activity (*a*_w_) and an exponential growth at high *a*_w_. In this study, the results were plotted on a moisture content dry basis.

All DFBG films presented type II isotherms, which indicated that the intelligent film incorporated with anthocyanin from DFSE had followed the GAB model. Therefore, it led to an excellent water‐binding capability and plasticizing effect on the DFBG films. A similar curvature was obtained when Vitis *amurensis* husk extract was added to intelligent films (Ma et al., 2017). Chi et al. (2020) produced κ–carrageenan‐based film incorporating grape skin powder and found that the moisture sorption of films increased at higher *a*_w_.

### 3.5 Effects of DFSE on Light transmittance of gelatin films

**FIGURE 3:**
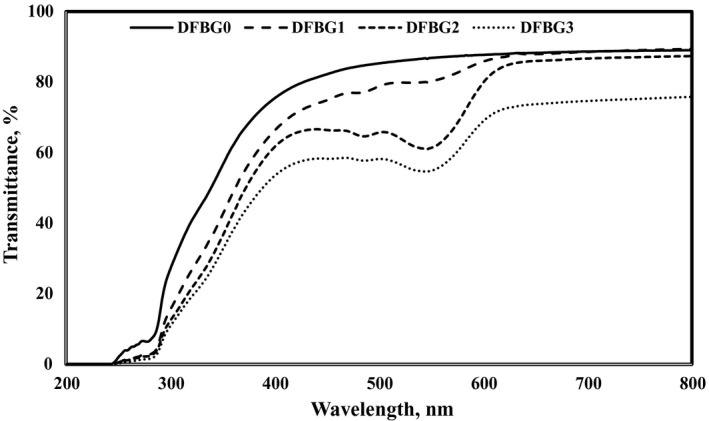
Effects of DFSE concentration on light transmittance of gelatin films

The light transmission properties of the films were obtained by UV–Vis spectrophotometry, as shown in Figure 3. According to Purbaningtias et al. (2017), UV–Vis light can accelerate food degradation and oxidation, leading to the loss of color of the film. In this study, the light transmission range was 200–800 nm, which DFBG0 film showed the highest transmission compared to other films. The UV–Vis light transmission of the films was found to decrease with increasing amount of DFSE in the films. DFBG3 film presented the lowest transmission (55.04%) due to unsaturated bonds in the anthocyanin that might have absorbed the UV–Vis radiation (Choi et al., 2017). Similar results were achieved when betalains from red pitaya peel were incorporated into starch/polyvinyl alcohol films (Qin et al., 2020).

The transmittance difference might be due to the color changes in the films, as shown in Table 2. The films showed reduced light transmission due to the impurities present in the DFSE residue, which had influenced the color of the films and the absorbance of the anthocyanin in the films (Choi et al., 2017). Hence, the higher addition of DFSE enhanced the light barrier property of the film. Zhang et al. (2019) revealed a decrease in the light transmission of gelatin‐based films with the addition of roselle anthocyanin in intelligent films.

**TABLE 2:** Color parameters of DFBG films after immersion in different pH buffer solutions

pH	Sample	Color attributes			Total difference in color
		L*	a*	b*	Δ*E*
**–**	DFBG0	95.65 ± 0.05^a^	−0.44 ± 0.01^a^	1.62 ± 0.02^a^	**–**
	DFBG1	94.42 ± 0.11^b^	1.30 ± 0.15^b^	3.22 ± 0.18^a^	2.15 ± 0.18^a^
	DFBG2	91.80 ± 0.08^c^	4.03 ± 0.07^c^	3.50 ± 0.01^b^	5.96 ± 0.07^b^
	DFBG3	87.83 ± 0.21^d^	9.98 ± 0.18^d^	4.31 ± 0.26^c^	13.16 ± 0.25^c^
pH 4	DFBG0	13.71 ± 1.25^a^	1.08 ± 1.26^a^	−0.64 ± 1.46^a^	–
	DFBG1	13.30 ± 1.01^a^	0.23 ± 0.30^a^	−0.46 ± 0.40^a^	2.47 ± 0.78^a^
	DFBG2	9.70 ± 1.94^b^	1.74 ± 0.95^b^	−0.98 ± 0.87^a^	4.87 ± 2.48^a^
	DFBG3	9.98 ± 0.40^b^	1.62 ± 0.11^a^	−2.27 ± 0.27^b^	4.28 ± 1.02^a^
pH 7	DFBG0	15.83 ± 1.44^a^	0.41 ± 0.32^a^	−0.80 ± 1.28^a^	–
	DFBG1	12.12 ± 1.94^a^	0.68 ± 0.75^a^	−0.86 ± 0.68^a^	4.95 ± 1.64^a^
	DFBG2	13.54 ± 1.73^a^	0.45 ± 0.33^a^	−1.90 ± 0.61^a^	3.24 ± 2.70^a^
	DFBG3	9.77 ± 0.76^b^	0.54 ± 0.43^a^	−2.42 ± 0.59^a^	6.46 ± 2.42^a^
pH 9	DFBG0	12.37 ± 0.79^a^	−1.23 ± 0.16^a^	−2.23 ± 0.37^a^	–
	DFBG1	10.90 ± 2.79^a^	−0.29 ± 0.18^ab^	−0.95 ± 0.98^a^	2.55 ± 0.85^a^
	DFBG2	9.26 ± 2.96^a^	−0.69 ± 0.43^a^	−1.19 ± 0.57^a^	3.33 ± 2.41^a^
	DFBG3	11.41 ± 1.65^a^	−0.63 ± 0.09^b^	−1.61 ± 0.40^a^	2.23 ± 0.58^a^

Note

Values are expressed as mean ± standard deviation. Values with the same superscript letter within a column are not significantly different (*p* > .05).

### 3.6 Colorimetric analysis and pH‐sensing evaluations

**FIGURE 4:**
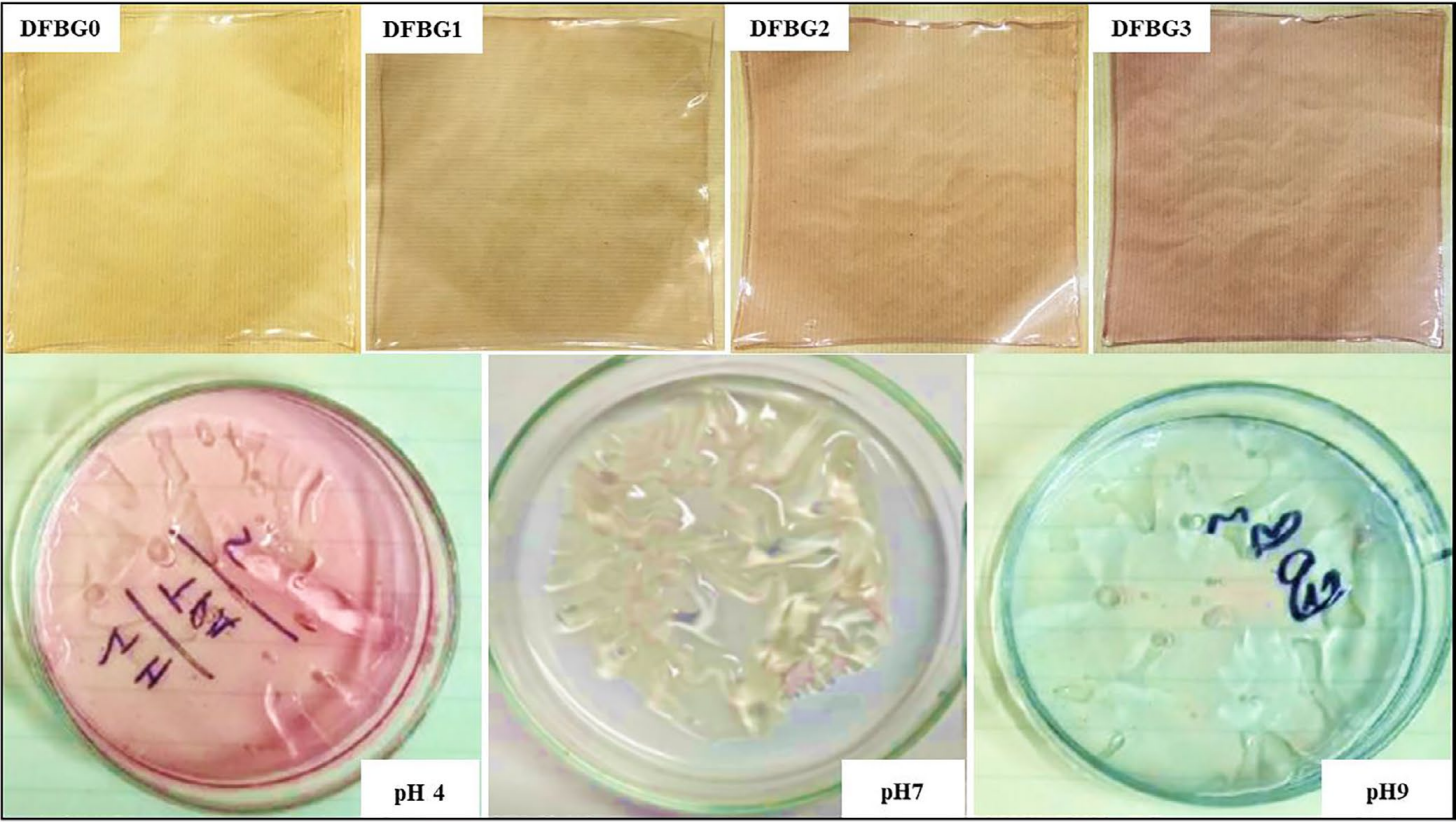
Color variations of pH‐sensitive films (DFBG0, DFBG1, DFBG2, and DFBG3) after drying and color changes in DFBG3 films after immersion in different pH buffer solutions

The color parameters of DFBG films after immersion in different pH buffer solutions is shown in Table 2. All the DFBG films were immersed for about 15 min before being analyzed for their color sensitivity. The color changes in DFBG3 films are shown in Figure 4.

The color characteristics of the intelligent film impact the appearance of the products. A good film can be achieved with increasingly bright colors produced by the film itself (Liu et al., 2017). All DFBG films exhibited a bright color, which is directly related to anthocyanin from DFSE.

The value of L* showed a significant decrease, indicating that an increasing amount of anthocyanin incorporated into the film has given brightness to the film. Meanwhile, a* value resulted in an increasing trend, which showed that the color changes to red. On the other hand, the decreasing trend can be seen in b* values that lead to the yellowish color of the films. DFBG0 film was found to be colorless since the L* value was the highest (95.65) while a* (−0.44) and b* (1.62) values were the lowest compared with the other films.

Besides, the highest value of Δ*E* (13.16), a* (9.98), and b* (4.31) can be seen from DFBG3 film with the lowest value of L* (87.83). The highest a* value obtained for DFBG3 film was due to the highest addition of anthocyanins into the intelligent film. Hence, the incorporation of DFSE has increased in a*, b*, and Δ*E* values, which agrees with the gradually deepened color of the films.

In an acidic medium (pH= 1–to 3), the anthocyanin usually appeared to be red which the flavylium cation be the predominant that changes the color to be red. In this study, pH 4 was used, meaning that pseudo‐base carbinol became predominant and it was colorless. However, the films produced low red color intensity, proving that a positive a* value was obtained.

Generally, in an alkali medium, the films turn green because the exposure of anthocyanins at high pH changes their structure, thus producing anhydrous bases (Araceli et al., 2009). A low‐intensity green color was detected after DFBG films were immersed in an alkali solution. The values of a* and b* were negative, indicating that DFBG films have greater blue color intensity than yellow color.

As the pH increased, the pH‐sensitive films presented negative chroma a* values. This revealed that red color has a higher intensity at low pH buffer solution while green color has a higher intensity at high pH buffer solution. The results show that incorporating anthocyanin from DFSE can contribute to film color changes and could be used as intelligent packaging.

Choi et al. (2017) demonstrated similar results. Films added with anthocyanin extract from purple sweet potato appeared to be green after being soaked at a higher pH buffer solution. The results are similar to a study conducted by Peralta et al. (2019) in which hibiscus extract was used in the polymeric films. According to Roobha et al. (2011), by addition of anthocyanin into the intelligent films changed to red color when it was subjected to the acidic medium.

### 3.7 Fourier transform infrared spectroscopy

**FIGURE 5:**
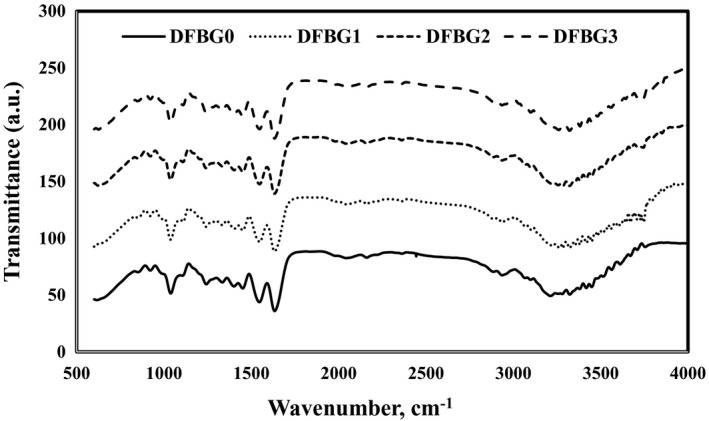
FTIR spectra of DFBG films

The FTIR spectra of all DFBG films are shown in Figure 5. The spectra of all films presented similar patterns, which indicate the similarity in structure.

In this study, DFBG0 film showed a robust and sharp band at 3610.74 cm^−1^ attributed to alcohol O‐H stretching in the films (Hanani et al., 2011). It also exhibited a functional group of amine N‐H stretching which can be seen from the band at 3448.72 cm^−1^ and 3323.35 cm^−1^. The bands at 3211.48, 2935.66, 2160.27, and 1633.71 cm^−1^ corresponded to alcohol O‐H stretching, alkane C‐H stretching, alkyne C≡C stretching, and alkene C=C stretching, respectively.

On the contrary, the incorporation of anthocyanin from DFSE had influenced the FTIR spectra. The robust broadband at 3448.72 cm^‐1^ was attributed to O–H of anthocyanin from the dragon fruit. The exact medium sharp bands at 3749.62 cm^‐1^ can be seen due to the presence of the alcohol group. The functional group was then recognized as O‐H stretching, which was contributed by the anthocyanin in the films. The addition of anthocyanin in intelligent films has shifted the wavenumber to be higher than the control film. According to Yousuf et al. (2016), this happened due to the chemical interaction between the aromatic rings of anthocyanin and the film matrix components. All DFBG films also showed the wavenumber of 1633.71 cm^‐1^, indicating alkene group C=C stretching.

Furthermore, FTIR spectra have shown that anthocyanin in intelligent films was immobilized in the film matrix because of the aromatic ring (Hanani et al., 2011). Choi et al. (2017) also presented similar results when O–H stretching band increased and shifted to a higher wavenumber when anthocyanin from purple sweet potato was added to agar/potato starch films. Recently, a similar result was achieved by Chi et al. (2020) who incorporated grape skin powder into κ‐carrageenan‐based intelligent films.

### 3.8 Scanning electron microscopy

**FIGURE 6:**
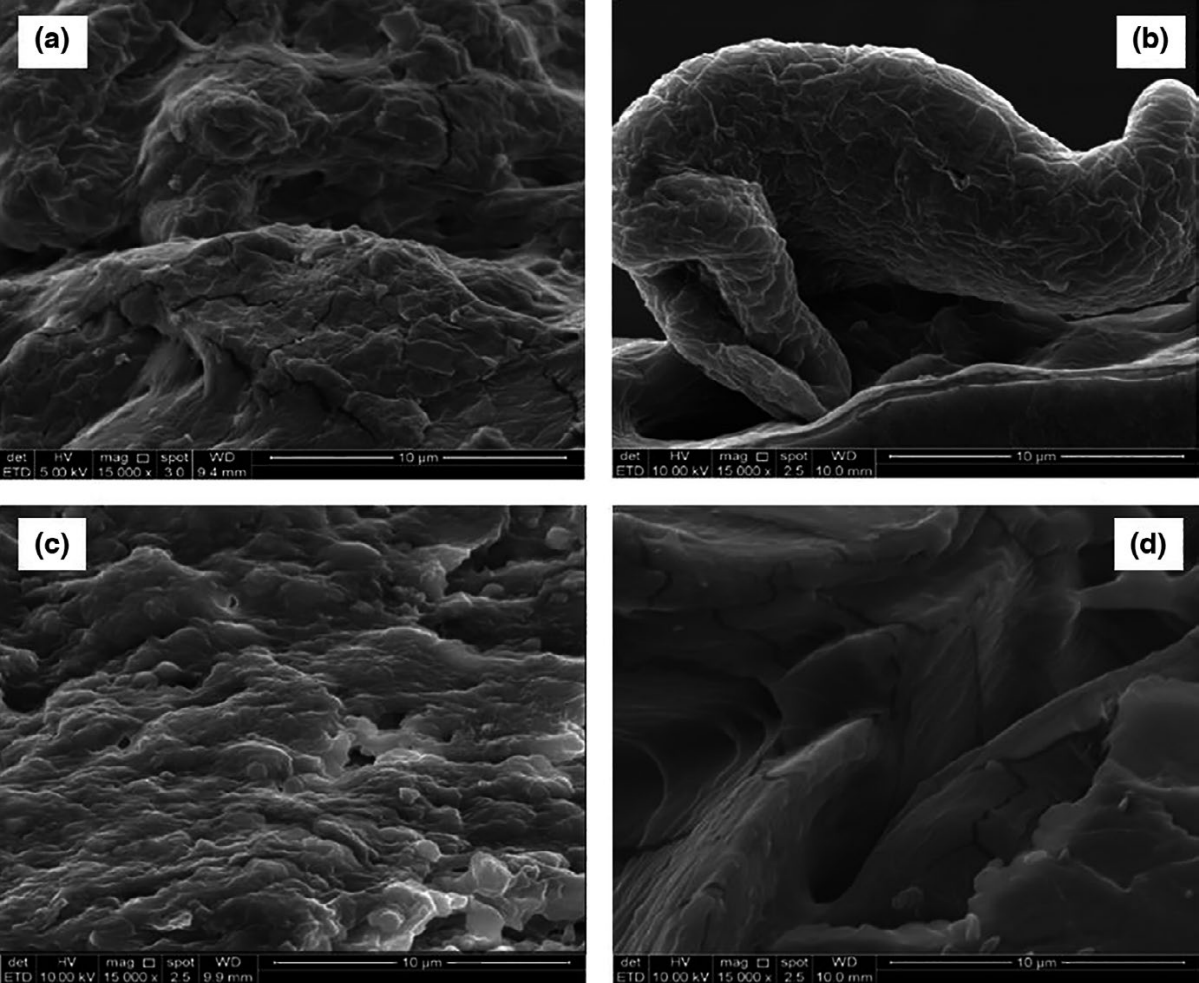
SEM images of the cross‐section of DFBG films. (a) DFBG0; (b) DFBG1; (c) DFBG2; (d) DFBG3

Scanning electron microscopy was used to study the morphological changes in the intelligent film incorporated with DFSE. Figure 6 shows the SEM images of the surface morphology of gelatin‐based intelligent film with anthocyanin from DFSE. A relatively rough surface can be seen in Figure 6a when there is no anthocyanin addition to the film preparation. Figure 6d indicates a DFBG3 film, which seems smooth and straightforward compared with the other films since anthocyanin in the film matrix might have influenced the network in the film matrix. Gelatin might also have contributed to the smooth surface. When DFSE was added to the film, the surface became smooth, suggesting that DFSE was compatible with the film components (Qin et al., 2020). Thus, it was proved that the DFSE could interact with major components in the film matrix through intermolecular interactions.

Akhtar et al. (2013) reported that the smooth film surface by incorporating beetroot and purple carrot extracts into the film matrix was due to the homogenous distribution of the extracts in the film matrix.

## 4 CONCLUSION

In this study, a new gelatin‐based intelligent film from the extraction of dragon fruit skin was successfully prepared. The addition of DFSE into DFBG films affected the films' mechanical properties, which reduced the tensile strength of DFBG films. Furthermore, according to the GAB model, all DFBG films presented sigmoidal curvatures; they have good water‐binding capability. The films showed that they could change their color (pink to green) over a wide pH range (pH 4–pH 9), with DFBG3 films found to be more suitable for application. Therefore, it can be concluded that the incorporation of DFSE into gelatin films has the potential to be used as intelligent packaging for the detection of freshness or spoilage of food products to ensure their quality and safety.

## CONFLICT OF INTEREST

The authors declare no conflict of interest.

## AUTHOR CONTRIBUTION

**Nurnabila Afiqah Azlim:** Data curation (equal); Formal analysis (equal); Investigation (equal); Writing – original draft (equal). **Abdorreza Mohammadi Nafchi:** Conceptualization (equal); Funding acquisition (equal); Project administration (equal); Supervision (equal); Writing – review & editing (equal). **Nazila Oladzadabbasabadi:** Investigation (equal); Project administration (equal); Visualization (equal). **Fazilah Ariffin:** Project administration (equal); Resources (equal); Validation (equal). **Pantea Ghalambor:** Formal analysis (equal); Validation (equal); Visualization (equal); Writing – original draft (equal). **Shima Jafarzadeh:** Data curation (equal); Investigation (equal); Project administration (equal). **A.A Al‐Hassan :** Formal analysis (equal); Funding acquisition (equal); Project administration (equal); Resources (equal); Software (equal); Validation (equal).

## ETHICAL APPROVAL

This study does not involve any human or animal testing.

## Data Availability

The data that support the findings of this study are available from the corresponding author upon reasonable request.
